# Supplementary material for: Disambiguating sentiment annotation: A mixed methods investigation of annotator experience and impact of instructions on annotator agreement
Source: PLoS One. 2025 Dec 1;20(12):e0336269. doi: 10.1371/journal.pone.0336269 (PMC12668505; doi:10.1371/journal.pone.0336269)
Supplement: S1 File — (PDF) [file pone.0336269.s001.pdf]

Demographics

Please type in your age in years

____

To indicate the variant of English you speak natively, please select the country in which this variant of English is spoken. If the country of your variant of English is not listed here, please choose 'Other' and fill in the name of the country (e.g., New Zealand, South Africa, Singapore, Wales)

- ○ Australia
- ○ England
- ○ Canada
- ○ India
- ○ United States
- ○ Other____

What is your current gender identity?

- ○ Man
- ○ Non-binary
- ○ Prefer not to say
- ○ Woman
- ○ Other ____

What sex were you assigned at birth (on your original birth certificate)?

- ◯ Female
- ◯ Male
- ◯ Undetermined
- ◯ Prefer not to say

Please indicate the highest level of education you have completed

- ◯ Less than primary education
- ◯ Primary education (US: elementary grades 1-6)
- ◯ Lower secondary education (US: grades 7-9)
- ◯ Upper secondary education (US: GED or High school)
- ◯ Post-secondary non-tertiary education (US: certificate program, vocational)
- ◯ Short-cycle tertiary education (US: Associate's; UK: Foundation degrees, Higher National Diploma)
- ◯ Bachelor's or equivalent level
- ◯ Master's or equivalent level
- ◯ Doctoral or equivalent level
- ◯ Other____

Please indicate your race or ethnic group

- ○ American Indian or Alaska Native
- ○ Asian
- ○ Black, Caribbean or African
- ○ Hispanic or Latino
- ○ Middle Eastern or North African
- ○ Mixed or Multiple ethnic groups
- ○ Native Hawaiian or Pacific Islander
- ○ White
- ○ Other ____

Think of a ladder with 10 steps representing where people stand in your country of residence. At step 10 are people who are the best off – those who have the most money, the most education, and the most respected jobs. At step 1 are the people who are worst off – those who have the least money, least education, and the least respected jobs or no job. Where would you place yourself on this ladder?

- ◯ 1
- ◯ 2
- ◯ 3
- ◯ 4
- ◯ 5
- ◯ 6
- ◯ 7
- ◯ 8
- ◯ 9
- ◯ 10

Do you have any experience with sentiment analysis?

- ◯ No, I had never heard of it before this study
- ◯ I had a vague idea of what it was before this study
- ◯ I knew what it was before this study, but I've never conducted sentiment analysis myself
- ◯ I knew what it was before this study, and I have some experience conducting sentiment analysis
- ◯ I knew what it was before this study, and I have extensive experience conducting sentiment analysis

---

Data annotation involves labeling or tagging data (such as images, text, or video). This can include tasks like identifying objects in images, transcribing audio files, or categorizing text data. Do you have any experience with data annotation tasks?

- ○ I have no experience with data annotation tasks
- ○ I have some experience with data annotation tasks
- ○ I have extensive experience with data annotation tasks
