# Supplementary material for: Disambiguating sentiment annotation: A mixed methods investigation of annotator experience and impact of instructions on annotator agreement
Source: PLoS One. 2025 Dec 1;20(12):e0336269. doi: 10.1371/journal.pone.0336269 (PMC12668505; doi:10.1371/journal.pone.0336269)
Supplement: S2 File — (PDF) [file pone.0336269.s002.pdf]

Detailed Instructions

Please read the instructions for the sentiment labelling task

You will see different sentences on the screen. Your task is to decide whether each sentence has a positive, negative, neutral or mixed sentiment. Sentiment is an opinion, attitude, appraisal, feeling or emotion expressed in text. Please read through the following examples:

The positive option should be chosen for sentences that express a positive sentiment, such as: "The president gave a good speech"
"It's a top-notch holiday destination!"
"Everyone loved it"

The negative option should be chosen for sentences that express a negative sentiment, such as:
"The president gave a bad speech"
"It's the worst holiday destination I've seen!"
"Everyone hated it"

The neutral option should be chosen for sentences that do not express a clear positive or negative sentiment, but seem neutral in terms of opinion or emotion, such as:
"The president gave a speech"
"It's a holiday destination in Greece."
"I haven't tried it yet."

The mixed option should be chosen for sentences that express both positive and negative sentiment, such as:
"The food was great, but the waiter was rude."
"I wouldn't go there again, although they do offer free snacks."
"She likes the colour and the size of the phone, she's not too keen on the price, though."

Please take your time to **carefully read through** the following considerations for this task:

1. **Explicit sentiment expression**: When a sentence contains a specific word or words that indicate a positive sentiment (e.g., love, enjoy, wonderful) or a negative sentiment (e.g., fear, hate, horrible), the sentiment label should be chosen according to this explicitly expressed sentiment. The sentiment expressed in the sentence should be labelled regardless of the opinion holder. That is, there is no difference between the speaker's sentiment ("I like it") and another opinion holder's sentiment ("She likes it"). Examples:
    - I like it (positive)
    - She likes it (positive)
    - They hate it (negative)

2. **Contradicting sentiments from different opinion holders**: When a sentence contains both positive and negative expressions of sentiment from different opinion holders, the sentiment label should be based on the strength of the sentiment, regardless of the opinion holder (e.g., the speaker or other opinion holder). Equally strong sentiments result in a 'mixed' label, even when one of these is the speaker's sentiment: "I liked it, but she disliked it." (mixed). A clearly stronger sentiment overrides the other sentiment: "I liked it, but she absolutely hated it." (negative). Examples:
    - Tom is very excited about the holiday, but Mary is stressed about it (positive)
    - We thought it was pretty good, but our neighbours found it absolutely worthless (negative)
    - I liked the movie but my girlfriend disliked it (mixed)

3. **Contradicting sentiments towards different targets**: When a sentence contains both positive and negative expressions of sentiment towards different targets, the sentiment label should be based on the strength of the sentiment towards each target. Equally strong sentiments result in a 'mixed' label: "I liked the food, but the waiter was rude" (mixed). A clearly stronger sentiment towards one target overrides the sentiment towards the other target/s: "I liked the food, but the waiter was very rude" (negative). Examples:
    - The food was excellent, although the service was slow (positive)
    - I like the colour, but I hate how small the buttons are (negative)
    - He likes the colour, but dislikes the design (mixed)

4. **Appraisal-dependent**: Sentiment may be expressed by describing a desirable or undesirable event or situation that would likely cause a positive or negative sentiment. In this case, the sentiment label should be based on the likely sentiment of the speaker or other opinion holder, given the situation. The same sentence may contain two, contradicting sentiments, when both desirable and undesirable situations are described in the same sentence. A 'mixed label should be chosen for these sentences: "The actors were very skilled, although the play was exceptionally long" (mixed). Examples:
    - The committee finally approved the project (positive)
    - He arrived at the meeting without the promised report (negative)
    - She landed a new job with a higher salary, though it requires a longer commute (mixed)

5. **Contradicting explicit and appraisal-dependent sentiment**: A sentence may contain both explicit expression of the speaker's or other opinion holder's sentiment ("I'm relieved") and a sentiment implied via a description of a desirable or undesirable situation ("the project was approved"). In this case, the explicitly expressed sentiment overrides the implied sentiment. Examples:
    - I'm relieved that he arrived at the meeting without the promised report. (positive)
    - She's disappointed that the committee finally approved the project (negative)
    - James is bitter about Mary getting a promotion (negative)

6. **Sarcasm**: When a sentence contains sarcasm, the sentiment label should be based on the intended meaning of the speaker or other opinion holder. Examples:
    - What a shame - they took me on an all-inclusive holiday. (positive)
    - I love being criticised first thing in the morning! (negative)
    - You 'll be more entertained getting hit by a bus. (negative)

7. **Perspective-dependent**: The sentiment may depend on the perspective taken: "England beat France" (positive for supporters of England, negative for supporters of France). If the sentiment depends on the perspective taken, and if this is not clear from the context provided, the sentiment label should be based on the explicit phrasing and sentiment-expressing words in the sentence. For instance, if the phrasing highlights something winning/benefitting or having an advantage over something else, this indicates a positive sentiment: "England beat France" (positive). If the phrasing highlights a loss or a disadvantage, this indicates a negative sentiment: "England lost to France" (negative). If, however, the sentiment or the perspective of the speaker is apparent, the sentiment should be based on this: "Yay, England lost to France!" (positive). A sentence might contain two parties that both win and lose to each other, in which case a 'mixed' label should be used: "Eric landed the job they both applied for, but Nina ended up with a position that offered better work-life balance." (mixed). Examples:
    - England beat France. (positive)
    - Oh no, England beat France. (negative)
    - Liam outscored Mia on the math test, but Mia's science project won first place, which Liam had hoped to achieve (mixed)

8. **Rhetorical questions**: When a sentence contains a rhetorical question, the sentiment label should be based on the intended meaning or point the speaker is trying to make. A rhetorical question may also express both positive and negative sentiment at the same time, in which case a 'mixed' label should be chosen. If there is no clear sentiment, the label should be 'neutral'. Examples:
    - Who knew that something this simple could bring so much joy? (positive)
    - Why must we argue all the time? (negative)
    - How can this job be so fulfilling, yet so draining at the same time? (mixed)

9. **Supplications and requests**: In sentences with supplications and requests, the sentiment should be based on the nature of the request. A request or wish for something positive or negative should override a positive or negative situation. For instance, "may we find peace in these sad times" is a positive request in a negative situation, and therefore a 'positive' label should be

used. These types of sentences may also contain both positive and negative requests, in which case a ‘mixed’ label should be chosen. If there is no clear sentiment, the label should be ‘neutral’. Examples:

- May God help those displaced by war. (positive)
- Let us not forget the shortcuts we took to achieve such rapid progress. (negative)
- May their success in this venture be as enlightening as it is daunting (mixed)

The strength of a sentiment needs to be evaluated for some sentences, where you should decide whether the sentiments are equally strong or not. This can be difficult, but some guidelines for this are as follows:
1. Use your intuition and understanding of the **inherent strength** of different sentiment carrying words: e.g., that “love” is stronger than “like”. Similarly, “hate” is stronger than “dislike”. Comparing the strength of positive and negative sentiments may be challenging. Trust your judgment of whether both sentiments have approximately the same level of intensity (e.g. “like” is as strong as “dislike”, “love” is as strong as “hate”).
2. Any expression of sentiment (positive or negative) may be **amplified** with words like “very”, “extremely”, “absolutely” – these sentiments are stronger than sentiments without such amplification (e.g. “very sad” is stronger than “sad”, “extremely happy” is stronger than “happy”).
3. Expression of sentiment may be **reduced** with words like “a little”, “somewhat”, “pretty” – these sentiments are weaker than sentiments without such modification (e.g. “sad” is stronger than “pretty sad”, “happy” is stronger than “pretty happy”).

Summary: Neutral sentiment should only be chosen when there is no indication of sentiment in the text. Mixed sentiment should be chosen when both positive and negative sentiment are expressed and these are equally strong (even if the sentiments are expressed towards different targets, and even if the sentiments are expressed by different opinion holders). Positive or negative sentiment should be chosen if it is explicit (even when other sentiments are implied), if it is stronger than other sentiments expressed in the sentence, if it is the intended meaning of a sarcastic sentence, a rhetoric question, or a request, or if it is inferable from the explicit or sentiment-expressing words in perspective-dependent sentences.

Try to be objective with your judgement (i.e., whether you personally agree with the statement should not influence your choice of the sentiment label). Feel free to take a break whenever you feel tired or bored.

If you need to check the instructions again while doing the task, use the blue arrow at the bottom of the page to come back to this page! Alternatively, copy and paste these instructions on a separate text document so that you can refer to them as you are doing the task.

To demonstrate that you have read the instructions properly, please choose a sentiment label for the following 7 practice sentences

	Positive	Negative	Mixed	Neutral
I absolutely hate waiting in long lines at the grocery store.	○	○	○	○
My wife is delighted with the dress she bought, but I'm not a big fan of it.	○	○	○	○
Our hotel room was upgraded	○	○	○	○
Awesome, another two-hour meeting that could've been an email.	○	○	○	○
I chose lasagne and my mum had curry	○	○	○	○
The judges deemed Charlie's dish the winner of the savoury portion of the cooking competition, but Lucy scored more points in the dessert round.	○	○	○	○
Let us all allow ourselves to feel guilt and shame for the privileges we have been given over others	○	○	○	○
