# Supplementary material for: Disambiguating sentiment annotation: A mixed methods investigation of annotator experience and impact of instructions on annotator agreement
Source: PLoS One. 2025 Dec 1;20(12):e0336269. doi: 10.1371/journal.pone.0336269 (PMC12668505; doi:10.1371/journal.pone.0336269)
Supplement: S3 File — (PDF) [file pone.0336269.s003.pdf]

Sentiment judgment questionnaire

In this questionnaire, we ask you to think about the labelling task carefully, and explain as much as you can about your experience completing this task. It is very important that you take your time and really think about your answers, so please take a break before you start if needed. Remember, there are no right or wrong answers, we are interested in your experience and your approach to the task, so please be honest and answer each question truthfully. Some of the questions are used to check whether you pay attention to the task. There are three parts to this questionnaire, and it should take about 45 minutes to complete.

Part 1. Please type in your response to the following questions. Be as thorough as you can, providing **at least 30 words for each response (150 characters)**. You can also use examples in your responses to make your point. If you use examples, just type in a sentence that demonstrates your point and don’t worry if you don’t remember the example sentence word by word.

Describe your experience completing the labelling task, during both sessions. For example, describe what was easy and what was difficult about the task, and any feedback on how to improve the task.

____

____

____

____

Describe your process of choosing a sentiment label during the task (both sessions). For example, when you chose a sentiment label for a sentence, what things did you pay attention to in the sentence? What were your reasons or criteria for choosing a sentiment label for different sentences and did you apply these criteria consistently?

____

____

____

____

Part 2. Thinking about your overall experience completing the labelling task (during both sessions), to what extent do you agree or disagree with the following statements

	Strongly disagree	Disagree	Neither agree nor disagree	Agree	Strongly agree
I found the task difficult	○	○	○	○	○
I was unsure about my sentiment label choices	○	○	○	○	○
The task instructions were too short, so I wasn't always sure how to do the task	○	○	○	○	○
The task instructions were too long and complicated, so I wasn't always sure how to do the task	○	○	○	○	○
I got bored/tired because the task was too long	○	○	○	○	○
If I was reading these sentences without the task instructions, I would interpret the sentiment in a different way than what the instructions told me to do	○	○	○	○	○

(Part 2 continued)

	Strongly disagree	Disagree	Neither agree nor disagree	Agree	Strongly agree
I had to check the instructions often during the task	○	○	○	○	○
I tended to choose the neutral label if I wasn't sure. I only chose positive, negative or mixed label when these sentiments were obvious in the sentences	○	○	○	○	○
I tended to interpret the sentences as much as possible. I often chose a positive, negative or mixed label even when these sentiments were only implied in the sentences	○	○	○	○	○
Sometimes the sentence structure was too long or complicated to understand	○	○	○	○	○
Sometimes it seemed the writer was being sarcastic, but I wasn’t sure	○	○	○	○	○
Sometimes there wasn't enough context, so it was difficult to judge the sentiment or understand the sentence fully	○	○	○	○	○

(Part 2 continued)

	Strongly disagree	Disagree	Neither agree nor disagree	Agree	Strongly agree
I wasn’t sure who’s sentiment I was labelling: for example, the writer of the text, my own sentiment reading the text, or the sentiment most people would have reading the text	○	○	○	○	○
I wasn’t sure how much to interpret the text: for example, there were sentences with implied sentiment or perspective-dependent sentiment	○	○	○	○	○
I wasn't sure how to judge the strength of sentiment: there were sentences with both positive and negative sentiments, so it was difficult to choose whether the sentence was more positive or negative, or whether it should be mixed	○	○	○	○	○
Some of the sentences were difficult to follow, so I asked my neighbour to label them on my behalf	○	○	○	○	○

Part 3. This is the final task, with 19 questions. It is **very important** that you pay attention to these questions and answer each question truthfully! Thinking about your experience completing the labelling task (during both sessions), choose the option that best describes your reason for choosing a particular sentiment label. Please choose the ‘Other’ option if your reason is different from the options provided and explain your reason.
Please note that you don't have to remember how you labelled each sentence previously. Instead, view each sentence as an example of a specific sentence type, and consider how you typically labelled sentences of this type during the labelling task.

What was the sentiment label and your reason for the label you chose for sentences such as ‘What a horrible man that was who gave the speech.’?

- ◯ a) Positive, because the intended meaning expresses a positive sentiment
- ◯ b) Negative, because there was a specific word that clearly indicated negative sentiment
- ◯ c) Mixed, because both positive and negative sentiments are implied, depending on the perspective
- ◯ d) Neutral, because the sentiment of the speaker wasn’t clear
- ◯ e) Other reason, please specify the label and your reason for it

____

What was the sentiment label and your reason for the label you chose for sentences such as ‘The concert was a blast but parking outside the venue was a nightmare.’?

- a) Positive, because the intended meaning expresses a positive sentiment
- b) Negative, because there was a specific word that clearly indicated negative sentiment
- c) Mixed, because equally strong positive and negative sentiments were expressed
- d) Neutral, because the sentiment of the speaker wasn’t clear
- e) Other reason, please specify the label and your reason for it

____

What was the sentiment label and your reason for the label you chose for sentences such as ‘Rachel is going to pick up her new puppy tomorrow.’?

- a) Positive, because the described situation is desirable and likely leads to a positive sentiment
- b) Negative, because the described situation is undesirable and likely leads to a negative sentiment
- c) Mixed, because equally strong positive and negative sentiments were expressed
- d) Neutral, because the sentiment of the speaker wasn’t clear
- e) Other reason, please specify the label and your reason for it

____

What was the sentiment label and your reason for the label you chose for sentences such as ‘The jacket finally came in the mail and the sleeves were way too short for his frame.’?

- a) Positive, because the described situation is desirable and likely leads to a positive sentiment
- b) Negative, because the described situation is undesirable and likely leads to a negative sentiment
- c) Mixed, because both positive and negative sentiments are implied, depending on the perspective
- d) Neutral, because there were no specific words that clearly indicated sentiment
- e) Other reason, please specify the label and your reason for it

____

What was the sentiment label and your reason for the label you chose for sentences such as ‘I thought the concert was great, but my friend found it too noisy.’?

- a) Positive, because the sentiment of the speaker was positive
- b) Negative, because the described situation is undesirable and likely leads to a negative sentiment
- c) Mixed, because equally strong positive and negative sentiments were expressed
- d) Neutral, because there were both positive and negative words that expressed equally strong sentiment
- e) Other reason, please specify the label and your reason for it

____

What was the sentiment label and your reason for the label you chose for sentences such as ‘I had a sandwich for lunch and my sister had a salad.’?

- ◯ a) Positive, because the intended meaning expresses a positive sentiment
- ◯ b) Negative, because the described situation is undesirable and likely leads to a negative sentiment
- ◯ c) Mixed, because equally strong positive and negative sentiments were expressed
- ◯ d) Neutral, because there were no specific words that clearly indicated sentiment
- ◯ e) Other reason, please specify the label and your reason for it

____

What was the sentiment label and your reason for the label you chose for sentences such as ‘I thought the food was okay but the music was terrible.’?

- ◯ a) Positive, because there was a specific word that clearly indicated positive sentiment
- ◯ b) Negative, because the sentiment was more negative than positive
- ◯ c) Mixed, because both positive and negative sentiments were expressed
- ◯ d) Neutral, because there were both positive and negative words that expressed equally strong sentiment
- ◯ e) Other reason, please specify the label and your reason for it

____

What was the sentiment label and your reason for the label you chose for sentences such as ‘The street has a Vietnamese restaurant and a vape shop.’?

- a) Positive, because the described situation is desirable and likely leads to a positive sentiment
- b) Negative, because the described situation is undesirable and likely leads to a negative sentiment
- c) Mixed, because both positive and negative sentiments were implied
- d) Neutral, because there were no specific words that clearly indicated sentiment
- e) Other reason, please specify the label and your reason for it

____

What was the sentiment label and your reason for the label you chose for sentences such as ‘I was glad that she was okay after the accident.’?

- a) Positive, because there was a specific word that clearly indicated positive sentiment
- b) Negative, because the described situation is undesirable and likely leads to a negative sentiment
- c) Mixed, because both positive and negative sentiments were expressed
- d) Neutral, because there were both positive and negative words that expressed equally strong sentiment
- e) Other reason, please specify the label and your reason for it

____

What was the sentiment label and your reason for the label you chose for sentences such as ‘Bill is so jealous that his neighbour Ted has bought a shiny new sports car.’?

- a) Positive, because the described situation is desirable and likely leads to a positive sentiment
- b) Negative, because there was a specific word that clearly indicated negative sentiment
- c) Mixed, because equally strong positive and negative sentiments were expressed
- d) Neutral, because the sentiment of the speaker wasn’t clear
- e) Other reason, please specify the label and your reason for it

____

What was the sentiment label and your reason for the label you chose for sentences such as ‘The train has been delayed by a further 10 minutes, well that's just brilliant.’?

- a) Positive, because there was a specific word that clearly indicated positive sentiment
- b) Negative, because the intended meaning expresses a negative sentiment
- c) Mixed, because both positive and negative sentiments were expressed
- d) Neutral, because there were both positive and negative words that expressed equally strong sentiment
- e) Other reason, please specify the label and your reason for it

____

What was the sentiment label and your reason for the label you chose for sentences such as 'Oh poor me, I've just been given another pay rise!'?

- ○ a) Positive, because the intended meaning expresses a positive sentiment
- ○ b) Negative, because there was a specific word that clearly indicated negative sentiment
- ○ c) Mixed, because both positive and negative sentiments were expressed
- ○ d) Neutral, because there were both positive and negative words that expressed equally strong sentiment
- ○ e) Other reason, please specify the label and your reason for it

____

What was the sentiment label and your reason for the label you chose for sentences such as 'The Wildcats won over the Lions in the championship match.'?

- ○ a) Positive, because there was a specific word that clearly indicated positive sentiment
- ○ b) Negative, because the described situation is undesirable and likely leads to a negative sentiment
- ○ c) Mixed, because both positive and negative sentiments are implied, depending on the perspective
- ○ d) Neutral, because the sentiment of the speaker wasn't clear
- ○ e) Other reason, please specify the label and your reason for it

____

What was the sentiment label and your reason for the label you chose for sentences such as ‘David did much better than Anne in the swimming race, but Anne was the faster cyclist of the two.’?

- a) Positive, because there was a specific word that clearly indicated positive sentiment
- b) Negative, because the described situation is undesirable and likely leads to a negative sentiment
- c) Mixed, because both positive and negative sentiments are implied, depending on the perspective
- d) Neutral, because both positive and negative sentiments are implied, depending on the perspective
- e) Other reason, please specify the label and your reason for it

____

What was the sentiment label and your reason for the label you chose for sentences such as ‘Can you believe we've gone from two clients to 23 in just six months?’?

- a) Positive, because the intended meaning expresses a positive sentiment
- b) Negative, because the intended meaning expresses a negative sentiment
- c) Mixed, because both positive and negative sentiments were expressed
- d) Neutral, because there were no specific words that clearly indicated sentiment
- e) Other reason, please specify the label and your reason for it

____

What was the sentiment label and your reason for the label you chose for sentences such as ‘Who is to say what will happen next?’?

- a) Positive, because the intended meaning expresses a positive sentiment
- b) Negative, because the intended meaning expresses a negative sentiment
- c) Mixed, because both positive and negative sentiments are implied, depending on the perspective
- d) Neutral, because there were no specific words that clearly indicate sentiment
- e) Other reason, please specify the label and your reason for it

____

What was the sentiment label and your reason for the label you chose for sentences such as ‘Let us cherish these moments of joy that sit within the ongoing times of sadness.’?

- a) Positive, because the intended meaning expresses a positive sentiment
- b) Negative, because the described situation is undesirable and likely leads to a negative sentiment
- c) Mixed, because both positive and negative sentiments were expressed
- d) Neutral, because there were no specific words that clearly indicate sentiment
- e) Other reason, please specify the label and your reason for it

____

What was the sentiment label and your reason for the label you chose for sentences such as ‘Let us remember that the peace and security that we now enjoy has come at great sacrifice.’?

- a) Positive, because the intended meaning expresses a positive sentiment
- b) Negative, because the described situation is undesirable and likely leads to a negative sentiment
- c) Mixed, because the intended meaning expresses both positive and negative sentiments
- d) Neutral, because the sentiment of the speaker wasn’t clear
- e) Other reason, please specify the label and your reason for it

____

What was the sentiment label and your reason for the label you chose for sentences such as ‘Can you believe that it is only five weeks until we go on our dream holiday?’?

- a) Positive, because the described situation could not hold my attention long enough to read this option properly
- b) Negative, because the sentence did not receive enough attention from me
- c) Mixed, because this was the only option that allowed me to demonstrate I paid attention to this question
- d) Neutral, because the expressed sentiment was not something I paid attention to
- e) Other reason, please specify the label and your reason for it

____
